# Bilateral anterior shoulder dislocations: A review of two cases and the relevant literature

**DOI:** 10.1002/ccr3.3351

**Published:** 2020-10-27

**Authors:** Malick Diallo, Massadiami Soulama, Delwendé Serge Romaric Kaboré, Patrick W. H. Dakouré, Philippe Liverneaux

**Affiliations:** ^1^ Service d’Orthopédie‐Traumatologie CHU Sourô Sanou (CHUSS) Nazi BONI University Bobo–Dioulasso Burkina Faso; ^2^ Service de chirurgie de la main Hôpitaux Universitaires de Strasbourg Strasbourg France

**Keywords:** acute, anterior, bilateral, chronic, epilepsy, shoulder dislocation

## Abstract

The bilateral symmetric anterior shoulder dislocations (BSASD) must be suspected in patients with bilaterally flattened shoulders after an uncontrolled muscular contraction like seizure condition. BSASD is best managed acutely and challenging to manage when diagnosed late. Chronic BSASD, after two years in a young patient, can result in fair functions.

## Graphical abstract

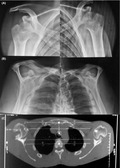

## 1 INTRODUCTION

Bilateral shoulder dislocations are a rare occurrence.^1^, ^2^, ^3^, ^4^, ^5^, ^6^ The two humeral heads dislocate from the scapular glenoid fossa in the same direction (bilateral symmetric shoulder dislocations or BSSD) or in different directions (bilateral asymmetric shoulder dislocations or BASD). There are three subtypes of BSSD (posterior,^1^, ^2^ anterior,^3^ and inferior^4^). BASD are classified as anteroposterior (one side anterior and the other side posterior)^5^ or anteroinferior (one side anterior and the other side inferior).^6^ Dislocations are acute or recent when recognized in the 21 days from the trauma. After 21 days, dislocations are called chronic or old.

Bilateral symmetric posterior shoulder dislocations are the most common type since Cooper in 1839^1^ and Myenter in 1902^2^ reported the first cases. The literature found less commonly bilateral symmetric anterior shoulder dislocations (BSASD).^7^

We report two cases of BSASD, one acute and one chronic case, both after epileptic seizures. We discuss the epidemiology, etiology, mechanism of injury, treatment, and outcome features through an extensive literature review of 133 BSASD reported cases.^3^, ^118^

## 2 CASE HISTORY

### 2.1 Case 1

**Figure 1:**
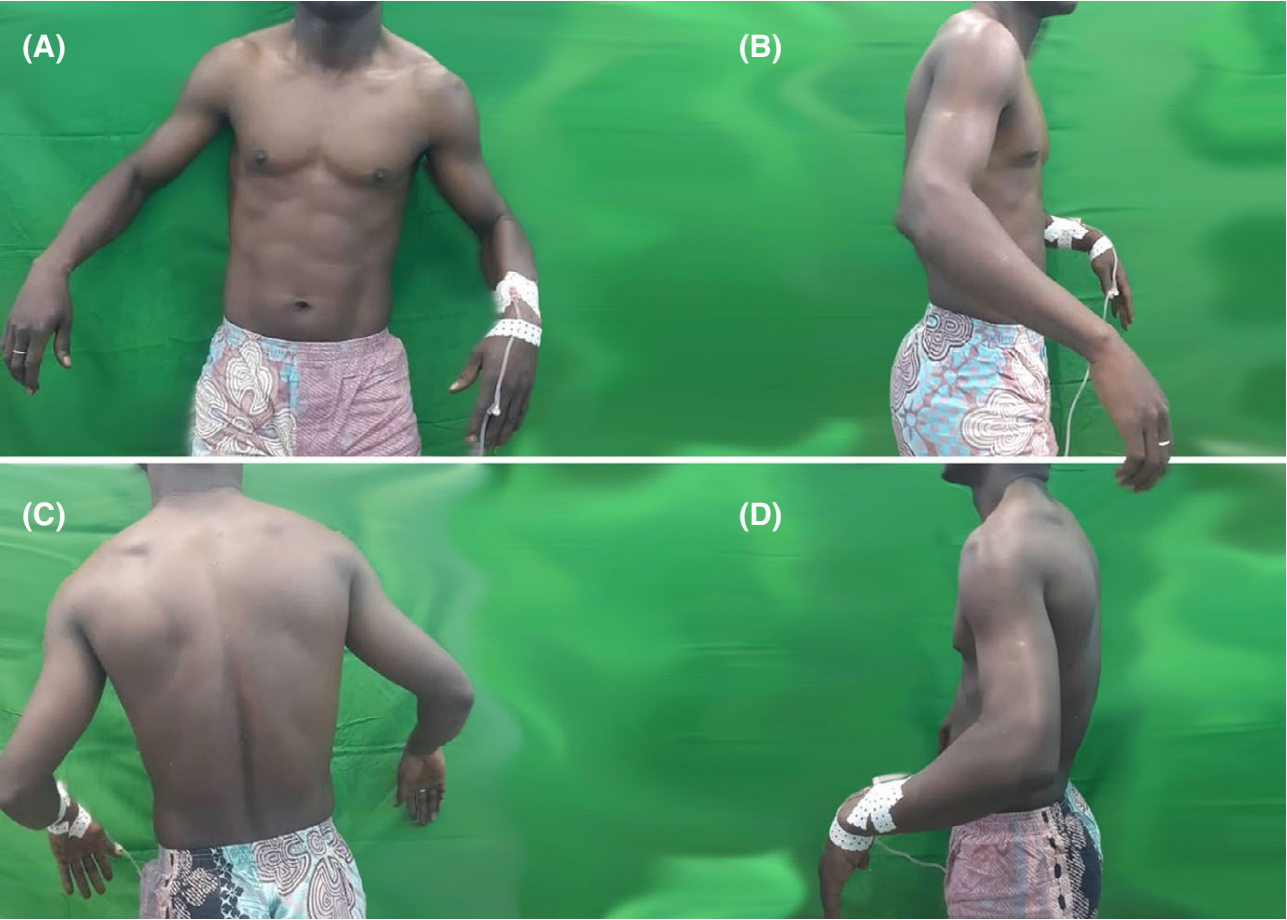
Clinical aspect of shoulders of an acute bilateral symmetric anterior shoulder dislocations in a 30‐year‐old man (Case 1) on the front (A), right‐side (B), back (C), and left‐side views. We noted bilateral square shoulders with arms fixed in abduction and external rotation

**Figure 2:**
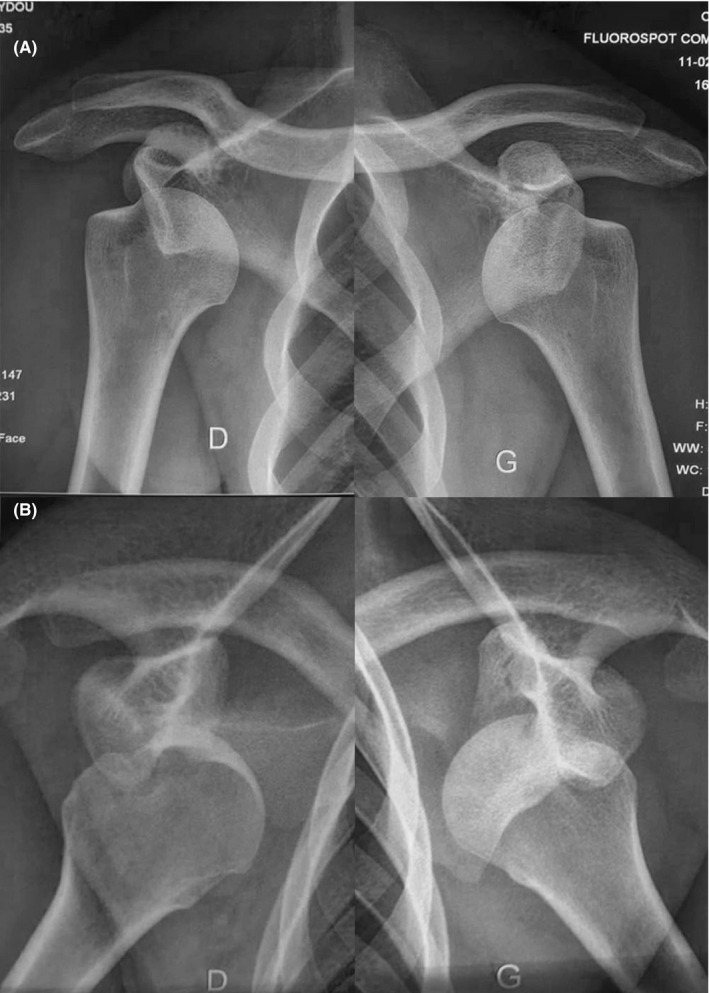
Shoulder radiographs (Case 1). AP (A) and Bernageau (B) views showed anteromedial subcoracoid displacement of both humeral heads

A 30‐year‐old male patient complained of bilateral shoulder pain at our ED. The patient woke up at night on the ground, a few minutes after falling from bed, but with no recollection of the fall. He had no prior history of epilepsy nor diabetes. An inaugural epileptic grand mal seizure was suspected. At admission, he presented with bilateral shoulder sulcus signs with an inability to rotate his arm internally (Figure 1). The neurovascular status was normal at both shoulders. Bilateral shoulder AP and Bloom‐Obata views revealed bilateral subcoracoid anteromedial glenohumeral dislocation without associated fracture (Figure 2). Closed reduction of both shoulders was performed under general anesthesia. With the patient in the supine position, we applied vertical traction and external rotation of the arm to achieve reductions. The postreduction evaluation demonstrated stable joints and normal neurovascular status. We performed strict immobilization with bilateral arm slings for three weeks. The patient was long lost of view. He was re‐evaluated 35 months later. At follow‐up, radiographs demonstrated concentrically reduced glenohumeral joints and painless full range of motion (ROM) at both shoulders with a Constant score of 98^1^.

**Table 1:** Bilateral symmetric anterior shoulder dislocation cases.^3^, ^118^ ^,^

Characteristic	Acute	Chronic	Total
Mean age (y)	40	41	40 (37.35‐43.87) CI 95%
Sex			
Male	77	15	92
Female	29	5	34
Indetermined	7	0	7
Mechanism of injury			
IMC‐associated	38	12	50
Traumatic	68	4	72
Nontraumatic	3	2	5
Indetermined	4	2	6
Associated injuries (right/left shoulder)	45/51	8/12	53/63
Great tuberosity fracture	22/21	6/8	28/29
3‐part fracture	6/4	0/0	6/4
4‐part fracture	1/3	0/0	1/3
Humeral neck fracture	0/1	0/0	0/1
Inferior glenoid rim fracture	0/1	0/0	0/1
Coracoid process fracture	3/2	0/0	3/2
Axillary vessels	2/2	0/0	2/2
Brachial plexus lesion	9/13	2/4	11/17
Long biceps tendon interposition	1/2	0/0	1/2
Rotator cuff tears	1/2	0/0	1/2
None	50	5	55
Indetermined	12	3	15
Treatment (right/left shoulder)	95/95	16/17	
None	0/0	3/3	3/3
Physiotherapy	0/0	0/0	0/0
Closed reduction	83/83	2/2	85/85
Open reduction	11/10	8/8	19/18
Arthroplasties	1/2	1/2	2/4
Refusal	0/0	1/1	1/1
Other (electrotherapy, Infrared …)	0/0	1/1	1/1
Indetermined	18	3	21
Mean follow‐up time (mo)	10.5	26.5	12.6
Instability	2/3	0/0	2/3
Recurrence	5/5	0/0	5/5
Range of motion (right/left shoulder)			
Full	60/60	7/8	67/68
Loss of motion	4/4	3/3	7/7
Not determined	49	9	58

Abbreviation: IMC, involuntary muscular contractions.

### 2.2 Case 2

**Figure 3:**
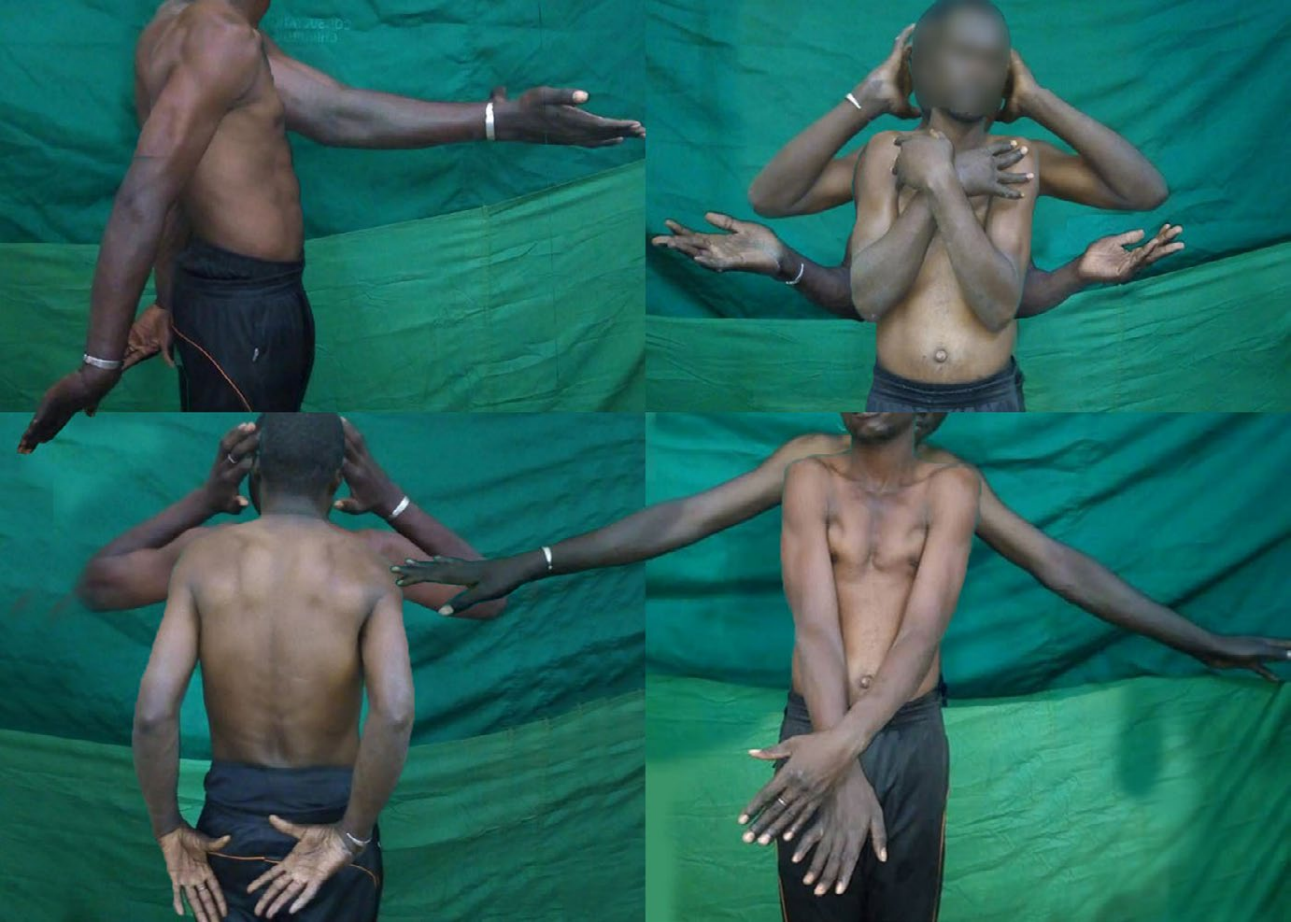
Range of mobility demonstrations in a chronic (2 y) bilateral symmetric anterior shoulder dislocations in a 26‐y‐old man (Case 2)

**Figure 4:**
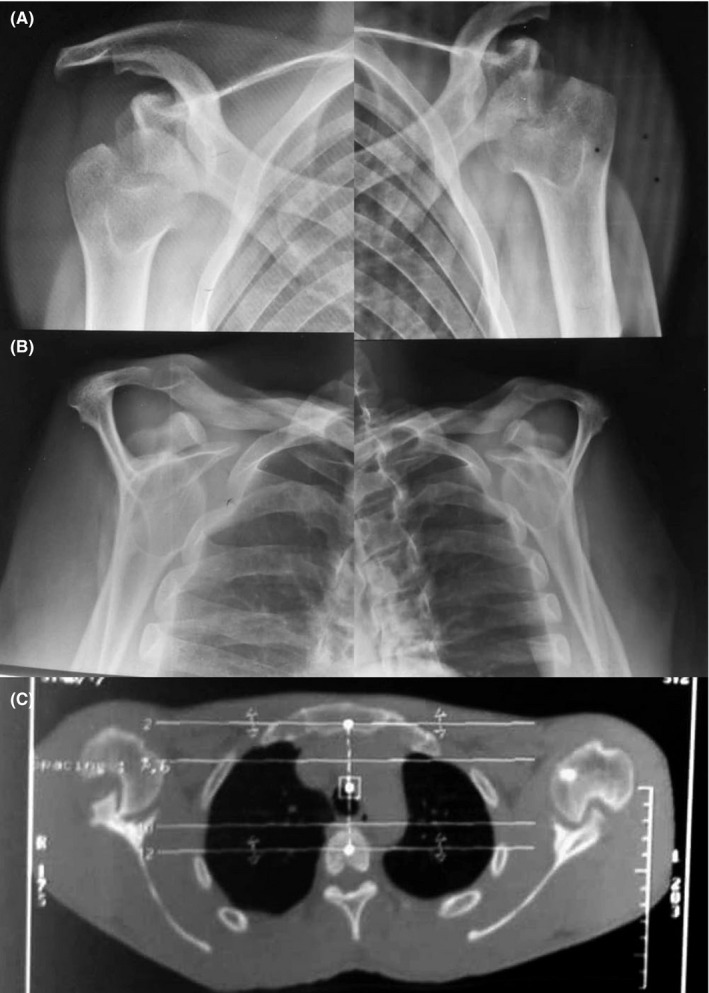
Shoulder radiographs and CT scan (Case 2). Between inferior glenoid rims and posterior Hill‐Sachs lesion (arrows) are arranged neoglenoid joints

A 27‐year‐old male farmworker presented two stiff shoulders, two years after an epileptic seizure. At the time of his initial injury, he was evaluated by traditional bonesetters who performed bilateral shoulder manipulations without anesthesia, after which he regained some mobility. For the subsequent two years, he was able to do some activities of daily living, including feeding, washing, and clothing himself. However, he was unable to do hard farming works. On our examination, he had bilateral shoulder sulcus signs with evidence of muscle atrophy in the scapular supraspinous and infraspinous fossae. Range of motion of the right shoulder was 85° of forwarding flexion, 30° of extension, 45° of abduction, 45° of cross‐body adduction, 15° of external rotation, and 25° of internal rotation. Range of motion of the left shoulder was 85° of forwarding flexion, 30° of extension, 85° of abduction, 45° of cross‐body adduction, 15° of external rotation, and 10° of internal rotation (Figure 3). The neurovascular status was normal. Medical imaging of shoulders included an AP and Bloom‐Obata view radiographs, and a CT scan. There was a bilateral subcoracoid anteromedial dislocation of the humeral head and an inverted neoglenoid‐like joint. The neojoint was arranged between the scapular inferior glenoid rim and a large Hill‐Sachs lesion (Figure 4). Despite poor Constant's scores (26.5/100 on the right side and 28.5/100 on the left), he was fully autonomous except for washing his upper back. We initially discussed an open reduction procedure. However, due to his successful adaptive shoulder function and his age, we recommended avoiding the surgery.

## 3 DISCUSSION

### 3.1 Epidemiology

From our analysis of the literature, the mean age of patients reported with BSASD was 40 years (SD = 18.43375) (Table 1). The youngest patient was 16 years old,^109^ and the oldest was 91 years old.^50^ The male‐to‐female ratio was 2.67. Due to pathognomonic "symmetric square shoulder aspect," referring to bilateral flattened shoulders, and inability to internally rotate either shoulder, dislocations are rarely missed in trauma condition. However, nontraumatic and involuntary muscular contraction (IMC) conditions can lead to missed dislocations.^9^, ^14^, ^18^, ^19^, ^21^, ^22^, ^31^, ^39^, ^41^, ^44^, ^64^, ^67^, ^83^, ^85^, ^95^, ^96^, ^100^, ^105^, ^106^, ^115^ Acute injuries represented 85% of BSASD reported in the literature. Twelve of the 20 chronic BSASD reported in the literature occurred in IMC conditions. The mean delayed time for chronic dislocations was 22 weeks (SD = 34.07323) after injury. Our second case presented later than any of the cases reported in the literature (two years). The delayed presentation was a result of belief in traditional bonesetters, poverty, and the acceptable adaptive function of shoulders. Patients rarely had previous unilateral^17^, ^56^, ^69^, ^114^ or bilateral^54^, ^82^ shoulder dislocations. Unilateral^17^ and bilateral^29^, ^54^, ^82^ recurrences were reported in traumatic cases.

### 3.2 Etiology and mechanism of injury

We sorted dislocations as traumatic, nontraumatic, and IMC‐associated.^7^

The mean age of patients with *traumatic BSASD* reported in the literature was 45 years (SD = 19.81311). We identified five main mechanisms: indirect force, indirect traction, indirect lever, direct force, and complex mechanisms.

- *The indirect force mechanisms* are mainly an anteroposterior directed force applied through the extremity (55.3%) with the shoulder in flexion, abduction, and external rotation (FABER). The classic circumstance is performing a bench press while weightlifting.^27^, ^32^, ^42^, ^46^, ^68^, ^69^, ^81^, ^91^, ^97^, ^116^ There is also the posterior‐to‐anterior force (39.5%) on the shoulder in hyperextension, abduction, and external rotation (EXABER). It commonly occurs when a patient is attempting to stop a backward fall from standing,^71^, ^87^ from a ladder,^29^, ^52^, ^98^, ^117^ a motorcycle,^63^, ^98^ and a two‐wheeled vehicle.^56^ Rarely, dislocations are reported when an inferosuperior directed force (5.2%) is applied to the shoulder in FABER, occurring when falling or diving in a pool.^33^, ^110^
- *The traction mechanism* is either a superior or anterior traction force applied through the extremity with shoulders in FABER.^11^, ^13^, ^66^, ^70^ Hanging on a riding horse,^66^ a motorcycle,^13^ and a curtain bar to prevent a fall^70^ were typical circumstances. One case of lateral traction in a quarrel has been described.^95^
- *The lever mechanism* was due to upper limb manipulations by bonesetters^94^ or by helping a paratrooper to remove his equipment.^111^
- *The direct hit* on shoulders is less common in BSASD.^22^, ^47^, ^55^, ^92^ A directed blow such as fall on the back^92^ or fall of a heavy object on a patient's back when leaning^55^ can drive the proximal humerus forward, resulting in a glenohumeral dislocation.
- *Complex mechanisms* associate two or more previously described mechanisms. They occur in high‐energy traumas such as a plane crash^9^ and a tractor accident.^22^

*Nontraumatic BSASD* is an exceptional condition. Inflammatory diseases such as rheumatoid arthritis^40^, ^44^ have been associated with glenohumeral subluxations and dislocations, likely due to substantial capsular and ligamentous destruction.

*IMC* causes most bilateral shoulder dislocations.^2^, ^3^ The review of IMC BSASD cases showed more males (4/5) with a mean age of 33 years (SD = 12. 53 966). Muscular spasms can be caused by an epileptic seizure as seen in our cases,^3^, ^17^, ^22^, ^24^, ^26^, ^30^, ^39^, ^51^, ^106^, ^107^, ^109^, ^113^, ^114^ an electrocution,^18^, ^20^, ^78^, ^85^ toxins (opiate, alcohol, chloroquine, and insecticide),^15^, ^23^, ^37^, ^104^, ^115^ a hypoglycemia,^28^, ^45^, ^54^ emotionally charged circumstances (nightmare and fear of death),^14^, ^67^, ^74^ or from the vibrations of a digging machine.^89^

### 3.3 Associated injuries

Forty‐three percent of BSASD were complicated, often bilaterally.

- *Associated fractures* accounted for two‐thirds of these complicating injuries. Isolated greater tuberosity (GT) fractures represent three‐quarters of fractures. Comminuted 3‐part and 4‐part proximal humerus fractures represented one‐fifth of fractures. GT and multipart proximal humerus fractures were present in traumatic and IMC dislocations with the same distribution. GT fractures were more common in traumatic indirect force dislocations^8^, ^34^, ^52^, ^63^, ^87^, ^99^, ^110^, ^112^ and complex proximal humerus fractures in direct hit and complex mechanisms.^55^ Nondisplaced associated coracoid process fractures seemed underestimated by the initial radiographs. Nondisplaced coracoid process fractures were often only found when early MRI was made.^45^, ^86^, ^101^
- *Associated neurovascular injuries* are less frequent (28.5%). When they do occur, the posterior and medial cords of the brachial plexus are often compressed,^22^, ^25^, ^52^ though sensorimotor function usually recovers with a good outcome. Four resolutions cases of axillary artery compression were reported in the literature.^11^, ^61^ Nervous status improves from week three and recovers in six months.^52^, ^70^, ^94^ Traction mechanisms put the highest risk on the neurovascular structures.^11^, ^91^ Neurovascular compressions must be recognized and addressed promptly to avoid sequelae.
- The others reported associated injuries are *rotator cuff tears* in GT‐free fractures,^29^ *the interposition of the long biceps tendon* in complex proximal humerus fractures,^34^ and *osseous Bankart lesions*.^37^

The chronicity enlarges the Hill‐Sachs notch as it becomes the neoglenoid fossa.

### 3.4 Treatment and outcome

Acute BSASD are mainly managed by closed reduction (CR).^7^, ^8^, ^13^, ^14^, ^17^, ^22^, ^32^, ^33^, ^35^, ^42^, ^45^, ^47^, ^48^, ^49^, ^50^, ^51^, ^52^, ^54^, ^56^, ^58^, ^60^, ^62^, ^63^, ^65^, ^66^, ^68^, ^69^, ^70^, ^71^, ^72^, ^74^, ^75^, ^76^, ^77^, ^80^, ^81^, ^82^, ^84^, ^86^, ^87^, ^88^, ^90^, ^91^, ^92^, ^93^, ^94^, ^96^, ^97^, ^98^, ^99^, ^101^, ^102^, ^103^, ^104^, ^107^, ^108^, ^109^, ^110^, ^111^, ^112^, ^113^, ^116^, ^118^ The presence of an associated lesion often necessitates operative management. Displaced GT fractures were managed by plate and screws with washers^96^, ^99^; anchors were used for rotator cuff tenodesis^99^, ^110^; and some authors performed primary Bankart‐like procedures.^102^ Complex fracture‐dislocations require extrication of the long biceps tendon^34^ and open reduction and internal fixation (ORIF).^34^, ^37^, ^45^, ^55^, ^73^, ^85^ Nagi et al^34^ choose primary hemiarthroplasty in a 4‐part proximal humerus fracture‐dislocation in a 49‐year‐old patient, though persistent neurovascular complications impaired the final outcome.^22^

In chronic BSASD, CR has been reported effective until week 4.^9^, ^20^, ^22^, ^64^, ^105^ Other authors have undertaken open reduction^95^, ^105^, ^115^ and associated anterior buttress^100^ or Bankart^106^ procedures with some good outcomes. In young patients, Hill‐Sachs lesions must be filled if the surgeon opts for an open reduction procedure. Bilateral hemiarthroplasty^41^ and reverse total shoulder arthroplasty^83^ should be proposed to patients older than 60 years. Like our second case, abstaining from surgery is a reasonable option for a young patient with satisfied adaptive functions.^83^, ^95^

## 4 CONCLUSION

The literature review showed sporadic cases of BSASD. Caution must be taken in epileptic and diabetes seizures to diagnose and treat patients promptly. Patient education, health insurance, and policy initiatives to improve access to health facilities are essential, especially in the low‐resource setting. It will avoid delayed presentation of chronic shoulder dislocations.

## CONFLICT OF INTEREST

All the authors declare that they have no conflict of interest.

## AUTHORS' CONTRIBUTION

DM: managed the patients, reviewed the literature, performed analyses, and wrote the manuscript. DSRK: managed the patients and performed the last evaluation. MS, PWD, and PL: reviewed the manuscript.

## ETHICAL APPROVAL

The patients were informed and consented to their data being collected and anonymized for education and scientific purpose. Unwritten verbal authorizations from both patients were obtained.

## Data Availability

The datasets used during the current study are available from the corresponding author on reasonable request.
